# Chloral Hydrate—Some of Its Uses*Read before the Philadelphia County Medical Society by Oscar H. Allis, M.D., for the author, June 13, 1894. This article Is not contributed exclusively to the *International Medical Magazine*, but has been sent at the same time to several journals for publication

**Published:** 1894-08

**Authors:** Ben. H. Broadnax

**Affiliations:** Broadnax, La.


CHLORAL HYDRATE—SOME OF ITS USES.*—In conversation with physicians at various times, I have noticed they viewed chloral as merely a hypnotic, and had used it only for the purpose of relieving pain, thereby inducing sleep. I have been a little surprised at this want of knowledge of its other equally valuable properties. Early in my practice I tried to make a few medicines, combined or by themselves, do all that they would for me, and was led into experimentation with them. Chloral came in for its share, because it relieved pain, quieted the nervous system, and did not paralyze the bowels.

As a *hypnotic*, five grains of chloral, combined with laudanum or with one-eighth or one-quarter grain of morphine, act splendidly, the combination intensifying the effects ef each and depriving the opiate of its stimulating property. With children, by itself, in sweetened water, it has no equal; mixed with paregoric, it is also good.

I prepare it as follows: I just cover the amount in my case vial with glycerin,—this dissolves it, and a drop is about a grain. In this form it mixes readily with oil or water and is more quickly prepared, and more easily divided into doses, large or small. With castor oil the dose, one to five grains, renders it less nauseating, and does not gripe, at the same time producing quiet and rest.

It is applied to the skin in eruptive diseases—measles, urticaria—as follows: chloral, ten grains (drops); carbolic acid, ten grains (drops); water or oil, one or two ounces; almost instant relief is experienced from the intense itchings. Or chloral, ten drops, with glycerin and water, each one-half ounce, produces the same effect.

As a mouth-wash: Chloral, ten grains, with glycerin and water, each one-half ounce (a teaspoonful), produces a pleasant, cool sensation in salivation, or as a gargle. After holding it for a moment in the mouth it should be rejected, and an equal amount of the fresh solution may be swallowed. Carbolic acid (ten drops) added makes it more effective in ulceration of the mucous coverings. It seems to act on the nerves locally, the same as chloroform by inhalation does on the body.

---

*Read before the Philadelphia County Medical Society by Oscar H. Allis, M.D., for the author, June 13, 1894. This article is not contributed exclusively to the *International Medical Magazine*, but has been sent at the same time to several journals for publication.

In toothache: Chloral, camphor, glycerin, carbolic acid, equal quantities, applied on a small piece of cotton after cleaning the cavity, will relieve the pain. (Cover with more cotton to fill the cavity.) I keep the mixture ready-made, under the name of "toothache drops," in my medicine case. If the patient has lost sleep I give a full dose of chloral by the mouth.

For ulcerated sore-throat, or ulceration from any cause, such as scalds: Chloral, ten to fifteen drops (grains); water, one to two ounces, as to age; sugar to make it palatable to children; a teaspoonful, repeated at short intervals, until sleep is induced, then on waking keep them fully under its influence. My first experience was on my only daughter, four years old. The case was so severe I feared I would lose her, and to get rest for her gave as above, after having tried everything else I knew of. The almost immediate relief of all the bad symptoms led me to think the medicine acted *otherwise than merely as a rest-producer.* Since then, for ten years, I have used it with the utmost satisfaction to myself and patients.

Earache: Camphor, ten grains; chloral, ten grains; carbolic acid, ten grains; castor oil, one-half ounce. Drop into the ear warm. Fill the ear full, apply a piece of cotton wet in warm water to fill the external ear, then a cloth wrung out in hot water as warm as can be borne. I have seen some children almost crazed go to sleep in two or three minutes and awake free of their troubles.

As an aid to chloroform in surgery or obstetrics ten to fifteen grains, given twenty minutes before administration of the anesthetic, seems to intensify the effect and less than one-half of it is needed to produce the desired effect. In my obstetric practice for the last fifteen years I have used it, and have observed but one case where any unpleasant effects were induced. This was in a woman with her tenth child. I gave the chloral to relax the system, ten grains; in half an hour five grains more; in half an hour the chloroform. This combination of drugs deadened her sensibilities so that she acted as if drunk and seemed incapable of ordinary movements. The birth of the child, however, was accomplished quickly and easily. She slept soundly for several hours and awoke all right. She was conscious and would answer questions, but her will-power seemed lost for awhile. This was the first time she had taken either of the drugs, and she may have been susceptible. Chloral, given before the anesthetic, seems to tide patients over the excited stage of anesthesia. The first few whiffs of the anesthetic produce quiet without any excitement. I have used it in a few surgical cases with the same effect. In children a full dose of chloral is administered, and when sleep comes on they are anesthetized in that state, and the force often otherwise necessary is thus avoided.

In coryza, where the Schneiderian membrane is very irritable, chloral, ten grains (or drops); castor oil, one-half ounce, used with a soft mop, applied over the surface, after being dried, acts to check the excretion of mucus, and lulls the irritation and the head-pains.

The supposed influence of the drug on the heart has been urged by my friends against its use. I have not seen any unpleasant effects. In any case where there is a chance of any cardiac trouble, it is an easy matter to fortify the heart with a one-fiftieth grain of nitroglycerin. In one delicate woman I did this as a precaution, but even in her case I believe it was not necessary. This summarizes my experience with chloral, and when I tell you I use from five to six pounds a year, you may know that it has a very considerable scope. I never prescribe it in any quantities, so as to create a "habit." In fact, I do not know of a single case of the kind.—*Ben. H. Broadnax, M.D., Broadnax, La., in Inter. Med. Magazine.*

